# Identification of Phenotypically Distinct Cancer Stem Cell Subpopulations in Oral Squamous Cell Carcinoma

**DOI:** 10.3390/cancers17213547

**Published:** 2025-11-01

**Authors:** Tarig Al-Hadi Osman, Oddveig Rikardsen, Muy-Teck Teh, Dipak Sapkota, Kristina Xiao Liang, Evelyn Neppelberg, Adrian Biddle, Ian Mackenzie, Lars Uhlin-Hansen, Anne Christine Johannessen, Daniela Elena Costea

**Affiliations:** 1 Department of Clinical Medicine, Faculty of Medicine, University of Bergen, 5020 Bergen, Norway; 2 Department of Medical Biology, Faculty of Health Sciences, University of Tromsø, 9010 Tromsø, Norway; 3 Department of Otorhinolaryngology, University Hospital of North Norway, 9019 Tromsø, Norway; 4 Centre for Oral Immunobiology & Regenerative Medicine, Institute of Dentistry, Barts & The London School of Medicine and Dentistry, Queen Mary University of London, London E1 4NS, UK; 5 Institute of Oral Biology, Faculty of Dentistry, University of Oslo, 0313 Oslo, Norway; 6 Department of Biomedicine, Faculty of Medicine, University of Bergen, 5007 Bergen, Norway; 7 Department of Oral Surgery, Haukeland University Hospital, 5009 Bergen, Norway; 8 Institute of Clinical Dentistry, Faculty of Medicine, University of Bergen, 5007 Bergen, Norway; 9 Centre for Cell Biology and Cutaneous Research, Blizard Institute, Barts & The London School of Medicine and Dentistry, Queen Mary University of London, London E1 4NS, UK; 10 Department of Clinical Pathology, University Hospital of North Norway, 9019 Tromsø, Norway; 11 Department of Pathology, Haukeland University Hospital, 5009 Bergen, Norway; 12 Center of Excellence for Cancer Biomarkers, CCBIO, Department of Clinical Medicine, Faculty of Medicine and Dentistry, University of Bergen, 5021 Bergen, Norway

**Keywords:** p75NTR, ALDH1, BMI1, cancer stem cell, tumor heterogeneity, plasticity, stochastic stem cell

## Abstract

Background: Several markers have been shown to define subpopulations enriched for cancer stem cells (CSCs) and correlate with poor clinical outcomes in oral squamous cell carcinoma (OSCC). Objective: To investigate the pattern of expression and correlation with clinical parameters of two CSC markers, namely p75NTR and ALDH1A1, in both patient samples and cell lines. Methods: Archival formalin-fixed paraffin-embedded samples from normal human oral mucosa (NHOM, n = 31), oral dysplasia (OD, n = 10) and OSCC (n = 177) were subjected to multiple immunohistochemistry and some to qRT-PCR for expression of CSC and proliferation-related markers, BMI1 and Ki67. Correlations between CSC marker expression and clinical parameters were investigated. Primary cells and cell lines derived from NHOM, OD or OSCC were FACS-analyzed for the same markers. Results: A higher frequency of cells positive for CSC markers was detected in OD and OSCC compared to NHOM. Co-localization of the two markers was a rare finding in OSCC as compared to NHOM or OD and was more heterogeneous in OSCC cell lines than in OD and NHOM cells. Cells positive for p75NTR exhibited higher expression of proliferative and self-renewal markers in comparison to ALDH1A1+ or double ALDH1A1+/p75NTR+ cells. Cells positive for p75NTR were more frequent in small-size tumors and poorly to moderately differentiated tumors and correlated with poor survival of patients otherwise (clinically) deemed as of better prognosis. Higher frequency of ALDH1A1+ cells was found to be associated with lymph node metastasis. Both p75NTR+ cells and ALDH1A1+ cells could emerge de novo from the respective negative subpopulation after FACS and in vitro growth, but with different kinetics. Conclusions: Here, we show that several stem cell subpopulations with distinct phenotypes co-exist in a tumor, each having impact on different clinical parameters. The cell subpopulations identified by the use of different CSC markers were found to be dynamic populations, able to switch between phenotypes. In addition, our data suggest that the stem cell heterogeneity is acquired and evolves parallel with carcinoma progression.

## Simple Summary

Cancer stem cells (CSCs) have been suggested to be the subset of cancer cells that drive tumor growth and progression and fuel the tumor with heterogeneous populations of cells, including those resistant to therapy. Different proteins have been proposed as biomarkers of CSCs in oral squamous cell carcinoma (OSCC). In this study, we simultaneously investigated multiple putative biomarkers of CSCs that have been previously proposed in OSCC, with the aim of identifying a pure CSC subpopulation. Our results suggest that several stem cell subpopulations may co-exist in a tumor, each having an impact on different clinical parameters. The cell subpopulations identified by use of different CSC markers were found to be dynamic and able to switch their protein markers over time. The inter-tumoral diversity of cancer stem cells demonstrated here raises novel challenges in terms of their use as predictive biomarkers and as efficient therapeutic targets for OSCC.

## 1. Introduction

The cancer stem cell (CSC) model for tumorigenesis [1] offers an explanation for some of the phenotypic and functional heterogeneity of cancer cells and may also elucidate some of the cancer-associated phenomena such as metastasis, therapy resistance and tumor recurrence [2]. Unlike the classical clonal evolution theory [3], the CSC theory proposes that tumorigenic ability is an exclusive characteristic of a subpopulation of cancer cells with stem cell-like properties. These cells give rise to progeny that generate the heterogeneous bulk of the tumor cells through differentiation and, hence, the hierarchical organization of a tumor [1]. Expression of pluripotency markers such as Oct3/4, Nanog and Sox2 by CSCs has been reported from several human tumors [4,5,6]. Nevertheless, identification of distinctive markers for distinguishing CSCs in a particular tumor type is still regarded as one of the key challenges in the CSC field [7]. Nevertheless, various markers for enriching for CSCs have been reported by studies of the same tumor type [reviewed in [2,7,8] and references therein], and therefore it has been speculated that the combinatorial use of sets of these markers might yield a purer CSC subpopulation [8].

Oral squamous cell carcinoma (OSCC), the most common type of head and neck cancer, with over 377,000 new cases worldwide in 2020 [9], remains an under-studied solid tumor. The conventional treatment approach of surgical resection followed by targeting rapidly proliferating tumor cells by radio- or chemotherapy results in only about half of the patients surviving beyond five years [10], which indicates the need for new treatment strategies. Immunotherapy for recurrent or metastatic cases has improved survival with few months, but only in a subset of patients [11]. The search for markers defining CSC in OSCC has also recently gained increasing attention, with several candidate markers reported [12,13,14,15]. The low-affinity nerve growth factor receptor p75NTR is a putative marker of normal oral and esophageal keratinocyte stem cells [16,17] and has also been suggested as a marker of CSCs in malignant melanoma and esophageal carcinoma [18,19], as well as a determinant of poor prognosis in OSCC [20,21]. Recent investigations performed by our group showed that the p75NTR+ OSCC subpopulation harbors cells with stem cell-like properties [22]. Aldehyde Dehydrogenase 1 (ALDH1) is an intracellular detoxifying enzyme with a well-established role in embryogenesis [23]. High activity of ALDH1 has been suggested to be a characteristic of both normal and cancer stem cells in the human colon [24] and breast [25]. ALDH1 activity has also been reported as a CSC marker in many other human cancers [26]. In OSCC, the isoform ALDH1A1 has been suggested as a CSC marker [12], and research has shown an overlap between ALDH1^High^ subpopulations and cells staining for CD44 [13], another marker generally accepted to identify and isolate a subpopulation of cells that is enriched for CSCs in OSCC [14,15]. Levels of the transmembrane glycoprotein CD44 have been repeatedly reported to predict poor patient prognosis, and cells expressing high levels of CD44 were shown to have higher invasion and migration abilities and higher expression levels of pluripotency markers than the bulk of the tumor cells [27,28,29]. In addition, several other biomarkers, such as CD133, CXCR4, ABCG2 and Musashi-1, have also been suggested as CSC markers in OSCC [30,31,32,33].

In the present study, we employed the triple immunohistostaining technique [34] to investigate the overlap of p75NTR and ALDH1A1-positive cell subpopulations in OSCC as compared to normal human oral mucosa (NHOM) and oral dysplasia (OD). Self-renewal and proliferation abilities of CSC-like subpopulations were also investigated in tissue samples by staining for BMI1 [35] and Ki-67 [36], respectively. The possible association between the frequency of positive cells, clinical parameters, and the overall survival of OSCC patients was investigated as well. Our results show marked heterogeneity in the expression, distribution, and overlap of p75NTR and ALDH1A1 among OSCC patients. The intra- and inter-tumoral phenotypic diversity of CSC-like subpopulations demonstrated here raises novel challenges in terms of their use as predictive biomarkers and as efficient therapeutic targets for oral squamous cell carcinoma.

## 2. Materials and Methods

### 2.1. Patient Material

Archived formalin-fixed paraffin-embedded (FFPE) tissues of patients with oral dysplasia (OD, n = 10) and OSCC (n = 177) were collected from Haukeland University Hospital and University Hospital of North Norway (UNN). Normal human oral mucosa tissues (NHOM, n = 31) were donated by patients having wisdom tooth extraction at Department of Oral Surgery, Haukeland University Hospital, Bergen, Norway, after informed consent. The samples from UNN were constructed in a tissue microarray (TMA) using Micro Tissue Arrayer (Beecher Instruments Inc., Sun Prairie, WI, USA). The tumor tissue was identified by comparing HE sections with the associated paraffin blocks. Two duplicate cores of 0.6 mm were taken from the representative tumor tissue and inserted into a recipient paraffin microarray block. The specimens were distributed on three different recipient microarray blocks. Paired material from lymph nodes positive for metastasis from 19 patients was also included.

All patients included in the study were newly diagnosed with SCC of the oral cavity and had no history of chemo- or radiotherapy prior to surgery. Tumors included in the study were from tongue, buccal mucosa, lip, gingiva, floor of mouth and alveolar rim. Tumors involving more than one of these sites were recorded as overlapping oral lesions. Sample collection was approved by the Research Ethics Council of North and West Norway (REK 2010/148). Material from 266 patients was initially selected, but 89 patients were later excluded from the study because they did not satisfy the above-mentioned inclusion criteria, or because of insufficient material or unavailable clinical data. The study followed the REMARK guidelines [37].

### 2.2. Triple Immunohistochemistry (IHC)

The following mouse monoclonal antibodies were used for IHC: anti-p75NTR (Merk Millipore, Burlington, MA, USA, Clone ME20.4, 1:2500), targeting the low-affinity nerve growth factor receptor located in the cell membrane; anti-ALDH1A1 (BD Biosciences, San Jose, CA, USA, Clone 44/ALDH, 1:250), targeting the enzyme aldehydedehydrogenase-1 isoform A1; anti-BMI1 (Merk Millipore, Clone F6, 1:1500), targeting the transcription factor polycomb complex protein BMI1, generally accepted as a marker of self-renewal; and anti-Ki67 (Dako, Carpinteria, CA, USA, Clone MIB-1,1:1500), targeting the proliferation marker mindbomb E3 ubiquitin protein ligase-1. Multiple immune-histochemical reactions combining three of the above antibodies were performed consecutively in each of the sections, as described elsewhere [34].

### 2.3. Evaluation of the Staining

Sections were evaluated using a light microscope equipped with X63 objective (Leica microsystems, Wetzler, Germany) and Zeiss digital camera operated by Zen 2011 software, version 1.0 (Carl Zeiss AG, Thornwood, NY, USA). For tumors in the tissue microarray, 2–4 cores were evaluated for each patient. The minimum number of cells accepted for a core was set at 300 cells. For the whole biopsy samples, four consecutive fields from the center of the tumor were evaluated, and when available, 2 fields from the para-tumor cancer-free surface epithelium (n = 62) and 2–4 fields from the invading front (n = 24). Nerve tissues and lymphocytes in the whole biopsy samples were used as internal positive controls for p75NTR and ALDH1A1/BMI1, respectively. Individual marker scoring was performed semi-quantitatively and scored into 4 levels as shown in Supplementary Table S1. Co-localization of all or pairs of the three markers was performed as shown in Supplementary Table S1, except for p75NTR+ALDH1A1+ which was scored as positive or negative. Inter-observer variation was controlled by calibrating the evaluation by three investigators (T.A.O., D.E.C. and A.C.J.). Afterwards, all samples were evaluated by one investigator (T.A.O.). The percentage of proliferating cells within p75NTR+, ALDH1A1+ and p75NTR+ALDH1A1+ subpopulations were obtained by counting Ki-67 positive and negative nuclei. No apparent difference in immunostaining was observed between the TMAs and the whole OSCC biopsy samples.

### 2.4. Laser Microdissection of FFPE OSCCs

Sections of FFPE were cut at 15 µm thickness, placed on membrane-coated glass slides (MembraneSlide NF 1.0 PEN, Zeiss) activated with UV light. Slides were incubated at 56 °C for 2 h, de-paraffinized in xylene, rehydrated in graded ethanol, stained with methylene green (S1962, Dako), and dehydrated in reverse graded ethanol and xylene. Tissue specimens 50–100 µm^2^ size were laser-microdissected from the tumor center and the corresponding invading front of each OSCC specimen using a Zeiss Axiovert 200 inverted microscope equipped with a microlaser system (P.A.L.M Microlaser Technologies). Microdissected tissues were collected in nuclease-free tubes (AdhesiveCap 500 clear, Zeiss).

### 2.5. Data Collection and Entry

Information collected for OSCC patients included age, gender, date of diagnosis, date of death or date of last follow-up, site of tumor, lymph node involvement, tumor size, TNM stage, recurrence of tumor, and tumor differentiation. No information regarding the medical history or sociodemographics was available for most of the NHOM donors.

For analysis, additional variables were generated from the information collected. Survival time was calculated as months from the interval between date of diagnosis and date of death or date of last appointment. Patients who were still alive at the time of investigation were recorded as “alive”, while patients who died for reasons other than cancer were censored, and only patients who died because of cancer were marked as “dead”. Another categorical variable was generated from survival time by grouping patients into those who survived five years or less, those who survived five to ten years, and those who survived more than ten years. Age was re-coded in decades, and lymph node metastasis and tumor recurrence as binary data (‘yes’ or ‘no’), while tumor size, TNM stage, and tumor differentiation were dichotomized (Supplementary Table S2).

**Figure 1:**
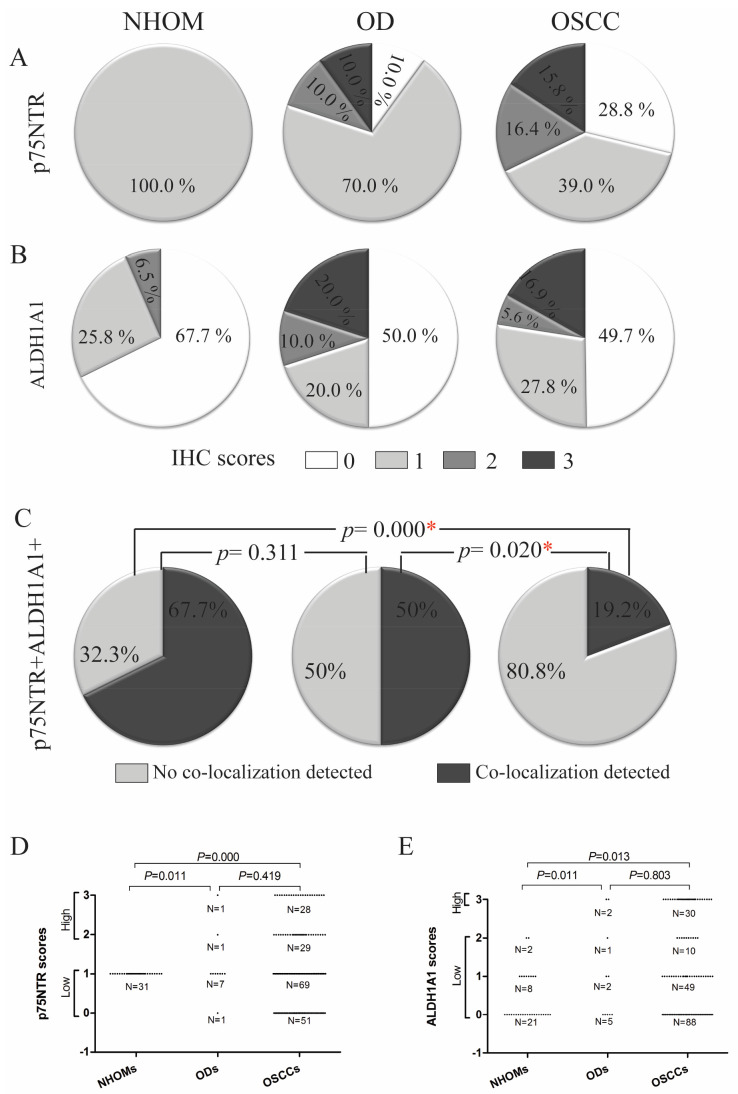
Frequency of samples positive for individual or combined CSC markers. Pie charts showing frequencies of crude single IHC scores of p75NTR (**A**) and ALDH1A1 (**B**) in NHOM, OD and OSCC. Comparison of distribution of dual CSC marker positivity in NHOM, OD and OSCC (**C**). statistically significant differences are indicated by *. Comparison of the distribution of dichotomized variables between NHOM, OD and OSCC (**D**,**E**).

**Figure 2:**
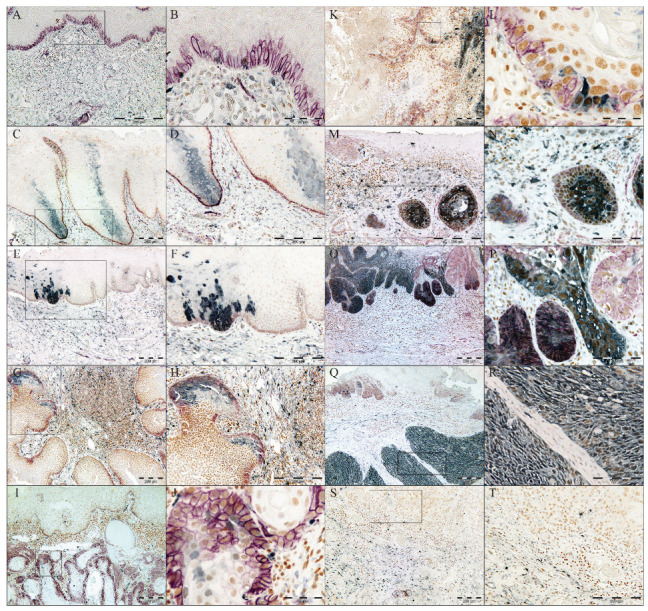
FFPE tissue subjected for triple IHC for p75NTR (purple), ALDH1A1 (grey) and BMI1 (brown) in NHOM (**A**–**D**), OD (**E**–**H**) and OSCC (**I**–**T**).

Individual IHC scores for each of the markers were dichotomized as ‘high’ or ‘low’, with the cut-off was determined according to the maximum score recorded for the respective marker in NHOM samples (Figure 1D,E and Figure 2B). Co-localization scores were dichotomized as ‘yes’ or ‘no’.

### 2.6. Cell Lines and Culture

A stepwise model of oral carcinogenesis consisting of a panel of cell lines was used in this study. As a model of OSCC, we used the following cell lines: CaLH3 [38], 5PT, Neo (known as PE-CA/PJ15) [39], LuC4 [38]; as a model of OD we used the following cell lines: DOK, Poe9n [40] and D20 [41]. As a model of normal oral keratinocytes (NOK), we isolated cells from NHOM donated by three patients who had their impacted wisdom tooth extracted at Haukeland University Hospital, after informed consent (REK2010/148). OSCC-derived cell lines, D20 and DOK, were grown in 3 to 1 DMEM/F-12 Ham, supplemented with 50 µg/mL L ascorbic acid, 0.4 µg/mL hydrocortisone, 10 ng/mL epithelial growth factor (EGF) (all from Sigma-Aldrich, St. Louis, MO, USA), 10% fetal bovine serum (FBS), and 5 µg/mL insulin (both from Life Technologies, Carlsbad, CA, USA), known in the literature as FAD medium [42]. NOK and Poe9n were grown in keratinocyte serum free medium (KSFM), supplemented with 10 ng/mL EGF and 25 µg/mL bovine pituitary extract (all from Life Technologies). All cells were grown under standard culture conditions.

### 2.7. Fluorescence-Activated Cell Sorting (FACS)

Cells were detached using 1X trypsin-EDTA (Sigma), re-suspended in PBS and incubated with mouse monoclonal anti-p75NTR antibody (Sigma, 1:250) for 5 min. Negative control mouse IgG1 (Dako) was used as isotype control. Alexa Fluor^®^ 488 F(ab^1^)^2^ fragment of goat anti-mouse H + L (Invitrogen, Carlsbad, CA, USA) was used as the secondary antibody. For the ALDEFLUOR^TM^ assay (STEMCELL, Cambridge, MA, USA), cells were incubated with the ALDEFLUOR^TM^ activated reagent with or without DEAB according to the manufacturer’s instructions prior to FACS. For both of the markers, the highest and lowest 4–5% cells were sorted, propagated under standard culture conditions and analyzed one week later.

To analyze cell lines simultaneously for the two markers, and also CD44, cells were stained for p75NTR first, using Alexa Fluor^®^ 647 donkey anti-mouse IgG (H + L) (Invitrogen) as secondary antibody. After washing and re-suspension, the cells were incubated for 5 min with PE-mouse antihuman CD44 (BD biosciences 1:1000), followed by the ALDEFLUOR^TM^ protocol.

All FACS procedures used a BD FACSAria^TM^ IIu (BD biosciences), using 525/50 BP filter for ALDEFLUOR^TM^ assay and Alexa Fluor 488, 660/20 for Alexa Fluor 647 and 575/26 for PE. Sorted cells were collected in FAD medium. Analysis of the results used Flowjo (version 10, Treestar, Ashland, OR, USA) or BD FACSDIVA^TM^ (BD Biosciences) (Supplementary Figure S1).

### 2.8. RNA Extraction, cDNA Synthesis and Quantitative RT-PCR (qRT-PCR)

Total RNA was extracted from p75NTR^High/Low^ and ALDH1^High/Low^ CaLH3 cells using RNeasy fibrous tissue mini kit protocol (Qiagen, Hilden, Germany). RNeasy FFPE Kit (Qiagen) was used to extract RNA from laser-microdissected tissues. Following the manufacturers’ instructions, 300 ng of total RNA was converted to cDNA using High-Capacity cDNA Archive Kit system (Applied Biosystems, Carlsbad, CA, USA). All qRT-PCR amplifications were performed on ABI Prism Sequence Detector 7900 HT (Applied Biosystems) as described previously [43]. qRT-PCR was used to examine the expression levels of the following genes (TaqMan assays): PDPN (Hs00366766_m1), NGFR (Hs00609947_m1 for RNA extracted from cells, and Hs00609978_m1 for laser-microdissected samples), BMI1 (Hs00180411_m1), CD44 (Hs01075861_m1), POU5F1(Hs 00999632_g1) and VIM (HS00185584_m1). GAPDH (Hs99999905_m1) was used as endogenous control. Comparative 2−ΔΔ Ct method was used to quantify the relative mRNA expression.

### 2.9. Statistical Analysis

Analysis of the qRT-PCR data was performed in GraphPad Prism 6 (GraphPad, San Diego, CA, USA) using the Student *t*-test, while all other statistical procedures were conducted in Statistical Package for Social Sciences (SPSS), version 19 (IBM, New York, NY, USA). Association between pairs of categorical variables was investigated by the Chi-Square test or Fisher’s Exact test. For comparison of immunohistochemistry scores for the tumor center and for the tumor invading front or the lymph node metastatic tumors, the McNemar test was used. Differences in percentages of Ki-67+ cells between different subpopulations in the same sample were investigated using repeated-measure ANOVA for normally distributed variables, while Friedman’s test was used for non-normally distributed variables. Further investigation between the same percentages within pairs of cell subpopulations was performed using Wilcoxon Rank test. Mean percentages of cells positive for different combinations of markers as obtained by multiple FACS analysis were compared between the three types of samples using Kruskal–Wallis test.

Survival analysis was investigated using Kaplan–Meier’s analysis. To control for possible confounding factors for patient survival, the analysis was repeated after splitting the data according to those factors. Moreover, Cox proportional hazards model was applied, and the impact of stem cell markers was evaluated by comparing the HR of the unadjusted analysis, to those adjusted for clinical or histological variables. All survival analysis procedures were performed in two different ways: analysis 1 included all study participants, while analysis 2 was performed on only patients who survived 10 years or less. In all statistical tests, the level of significance was set to 0.05.

## 3. Results

### 3.1. Clinical and Pathological Characteristics of the Study Cohort

The age of the patients included in this investigation (n = 177) ranged from 27 to 93 years (mean ± SD 65.31 ± 12.92 years). The patients were predominantly males (61.5%), older than 60 years old (62.1%), diagnosed with late-stage tumors (56.3%) mainly occurring in the tongue (52.0%). Survival time of the patients ranged from 1 to 295 months (64.08 ± 62.42) from the date of diagnosis. Higher survival probabilities were observed for patients aged ≤60 years (*p* = 0.001), diagnosed with stage 1 and 2 (*p* = 0.000) or with highly differentiated tumors (*p* = 0.012), as compared to those older than 60 years old, with late-stage or poor to moderately differentiated tumors, respectively, using Kaplan–Meier analysis (Supplementary Figure S2). Further details about the clinical information of the study participants are presented in Supplementary Table S2.

### 3.2. Increased Frequency of p75NTR+ and ALDH1A1+ Cells in OD and OSCC Compared to NHOM

In all NHOM samples, p75NTR was visualized by IHC as a membranous staining of groups of epithelial cells (less than 10% of the total cell number) confined to the basal layer (Figure 1A and Figure 2A,B, Supplementary Figure S3A). The same pattern was observed in 70% of the OD samples, with the rest displaying an increase in the frequency of cells expressing p75NTR (Figure 1A and Figure 2E, Supplementary Figure S3A). The heterogeneity of P75NTR staining pattern further escalated in OSCC (Figure 1A), with only 9% showing a preserved NHOM pattern (Figure 2G,H), while 15.8% of OSCCs contained more than 50% p75NTR+ cells (Figure 2O,P), and interestingly, 28.8% of OSCC showed loss of P75NTR staining (scoring less than 1% or being completely negative for p75NTR+ in the epithelial compartment). It is noteworthy that in the latter case, positive staining of nerves ruled out technical issues in detecting p75NTR (Figure 2S,T). Distribution of p75NTR+ cells was predominantly in less differentiated tumors or the peripheral basaloid cell layer of the more differentiated tumor islands (Figure 2I–N). Importantly, detection of p75NTR+ cells in the more differentiated central parts occurred only in samples with high frequency of p75NTR+ cells (Figure 2O,P).

ALDH1A1 was detected as a cytoplasmic IHC reaction with occasional nuclear involvement, in sporadic cells of the basal and spinous cell layers of NHOM (Figure 2A,B), with the exception of two samples where ALDH1A1+ cells were detected as a vertical patch of stratified keratinocytes (Figure 2C,D). Expression of ALDH1A1 was generally low in NHOM samples, with 67.7% of samples displaying less than 5% ALDH1A1+ cells (Figure 1B, Supplementary Figure S3B), and among these, 8 samples (25.8%) showed no ALDH1A1+ epithelial cells at all. Higher (5–25%) expression levels of ALDH1A1 were found to be infrequent in NHOM (Figure 1B and Figure 2C,D). The OD samples showed a wider range of scores for the frequency of ALDH1A1+ cells, with two samples expressing it in more than 50% of the cells (Figure 1B, Supplementary Figure S3B). In OSCC, the frequency of ALDH1A1+ cells was found to be even more heterogeneous than in OD (Figure 1B, Supplementary Figure S3B), with tumor islands showing more than 50% ALDH1A1+ cells being detected next to islands scoring 0% (Figure 2K–R).

The frequency of dichotomized IHC scores of p75NTR and ALDH1A1 was significantly different between NHOM samples and OD and OSCC samples (Figure 1D,E). The two factors were colocalized more frequently in NHOM (67.7%) and OD (50%) than in OSCC (19.2%) (*p* < 0.001, Chi-Square) (Figure 1C). Co-localization was primarily observed in samples with high expression of both markers, although it could be detected even when one or both markers were expressed at very low levels (<1% positive cells for p75NTR and <5% positive cells for ALDH1A1) (Supplementary Figure S3). No significant association was found between presence of double-stained cells and any of the clinical parameters examined.

### 3.3. Identification of a Pattern Resembling a Clone-Like Distribution of ALDH1+ Cells

In the two NHOM samples (6.5%) that had higher frequency of ALDH1A1 cells (25–50%), the ALDH1A1 was detected as a clone-like pattern extending from the basal layer and involving 3–4 layers of the spinous cell layer (Figure 2C,D). This distribution pattern of ALDH1A1-expressing cells was also detected in 30% of OD cases (Figure 2E,F), and in the para-tumor epithelium of 56.6% of the OSCC samples that included such areas (n = 62). This distribution pattern was found not to be associated with any of the clinical parameters included in the analysis but indicates that some of the more differentiated epithelial cells can express ALDH1A1.

### 3.4. Frequency of p75NTR+ Cells in OSCC Cells Predicts Survival in Patients with Small Tumor Size (T1 and T2)

High frequency of p75NTR+ cells is inversely correlated to tumor differentiation and tumor size (*p* = 0.034 for both, Chi-Square). No association was found between p75NTR frequency and tumor recurrence, lymph node metastasis or TNM stage. A high frequency of ALDH1A1+ cells was found to be associated with lymph node metastasis (*p* = 0.035, Chi-Square test) (Supplementary Figure S4).

**Figure 3:**
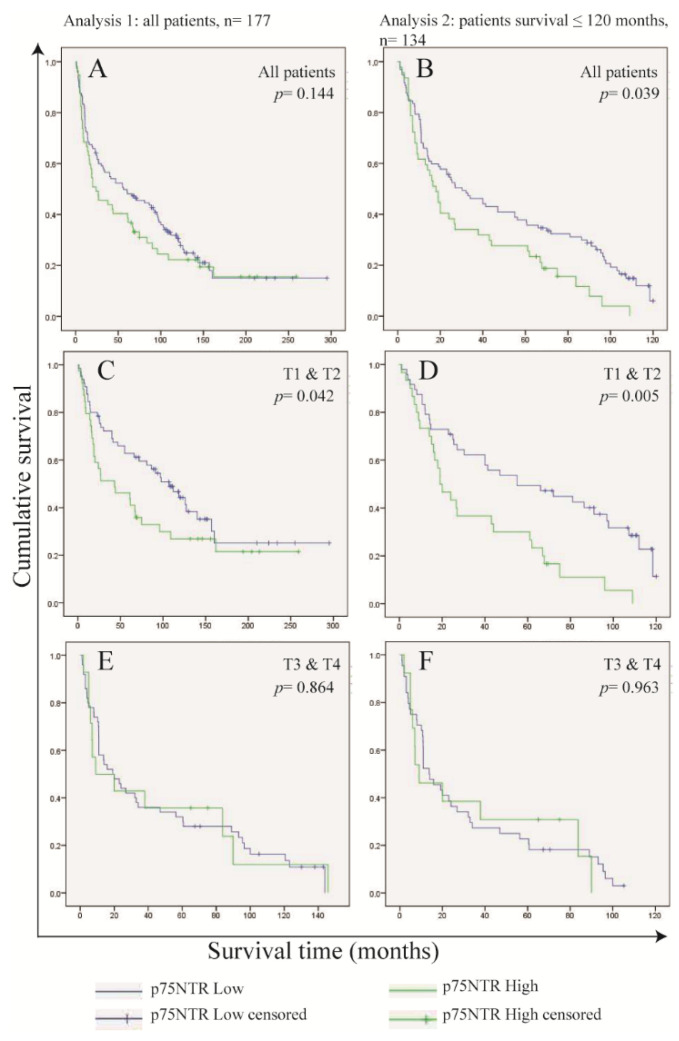
Expression of p75NTR predicts survival in patients with tumors of small size. Kaplan–Meier’s curves including all patients (**A**,**B**), patients with small-sized tumors (T1 and T2 in (**C**,**D**)) and large-sized tumors (T3 and T4 in (**E**,**F**)), illustrating differences in survival probabilities between cases with high and low p75NTR expression. The analysis was performed for the entire cohort (left panels), and separately for patients who survived ≤10 years (right panels).

Kaplan–Meier’s analysis indicated a lower survival probability of patients whose tumors were found to express high levels of p75NTR, but without statistical significance (0.144, Tarone-Ware), and the survival probabilities were similar after 120 months from the date of diagnosis (Figure 3A). Comparing the characteristics of patients who survived beyond 120 months (n = 33), we found that most of these patients were diagnosed at ≤60 years (69.7%), with T1 and T2 tumors (61.9%), no lymph node metastasis (72.7%) at the time of diagnosis and no tumor recurrence afterwards (81.8%) (Supplementary Table S2, Supplementary Figure S2). Excluding these patients from the analysis revealed a statistically significant difference (0.039, Tarone–Ware) in the survival probabilities between patients with p75NTR^High^ tumors and those with p75NTR^Low^ tumors (Figure 3B).

Knowing that high expression of p75NTR was associated with small-size tumors, we performed Kaplan–Meier’s analysis by stratifying for tumor size, and we found p75NTR of predictive value only in patients diagnosed at T1 and T2 (0.042, Tarone–Ware) (Figure 3C–F). Therefore, we investigated the role of tumor size as a confounding factor by Cox regression hazard models (Supplementary Table S3). The models showed a marked reduction in the hazard ratio of p75NTR when adjusted for tumor size. This was observed when the models were applied to the whole study group (analysis 1), as well as when applied to patients who have survived 120 months or less (analysis 2). However, statistical significance of the interaction term between p75NTR expression and tumor size was only observed in analysis 2.

These data show that the effect of p75NTR in patient survival is of importance in patients with tumors of small size.

### 3.5. Highest Expression of Self-Renewing Marker BMI1 Was Detected in OD

**Figure 4:**
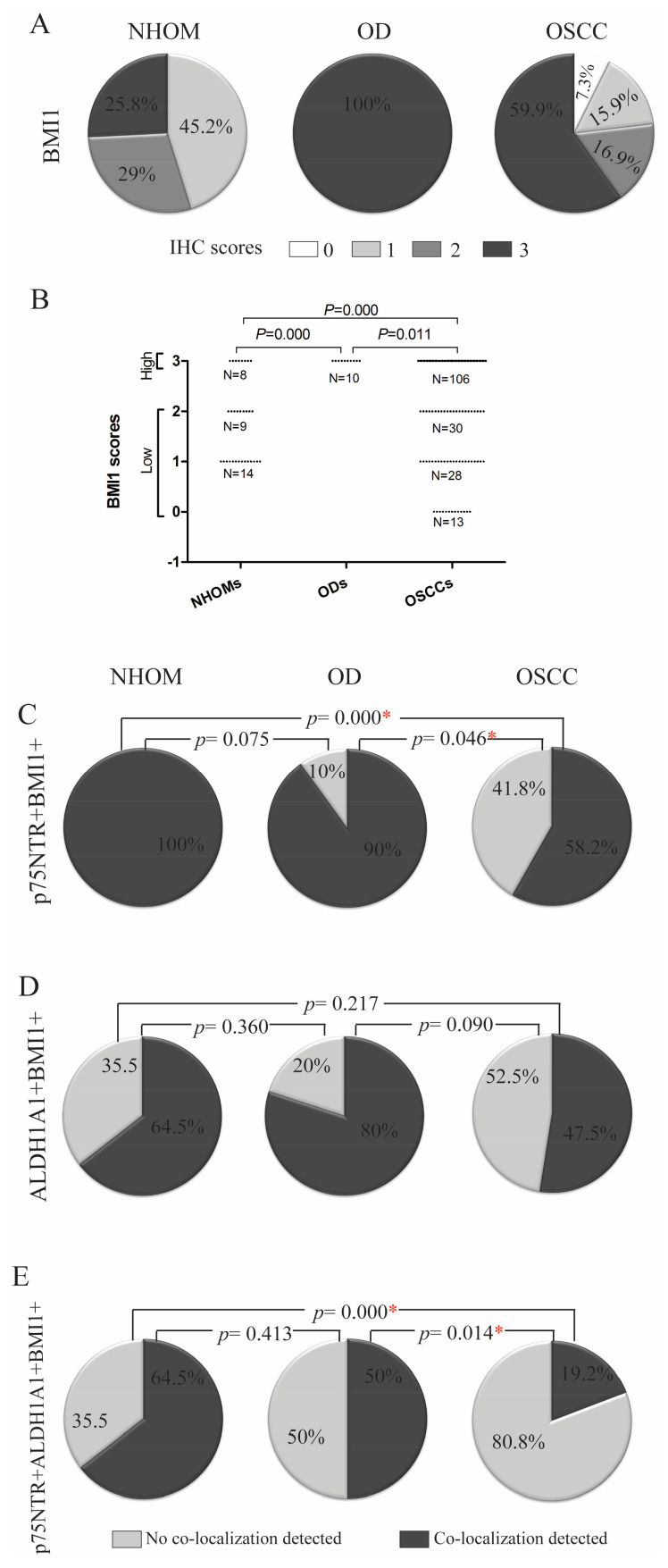
Comparison of the crude (**A**) and the dichotomized (**B**) IHC scores of BMI1 (**A**) in NHOM, OD and OSCC. Comparison of the frequency of p75NTR+BMI1+ (**C**), ALDH1A1+BMI+ (**D**), p75NTR+ALDH1A1+BMI1+ (**E**), between the three types of samples. Statistically significant differences are denoted by *.

Expression of the self-renewal marker BMI1 was quite frequent in all samples examined. BMI1 exhibited nuclear localization and was detected both in the basal and spinous epithelial layers across all NHOM samples, with a high frequency of positive cells (>50%) observed in 8 samples (25.8%) (Figure 4A). High expression of BMI1 was observed in all OD samples (Figure 4A). In OSCC, the expression of BMI1 was variable, with only 59.9% (n = 106) of the samples displaying high frequency of BMI1+ cells, while 7.3% (n = 10) displayed less than 1% BMI1+ cells (Figure 4A). BMI1 expression was found to be significantly higher in OD as compared to both NHOM and OSCC samples (Figure 4B, Supplementary Figure S3C). The BMI1 score did not correlate with any of the clinical parameters investigated.

### 3.6. A Greater Number of p75NTR+ Cells Co-Expressed BMI1 Compared to ALDH1A1+ Cells

An overlap between p75NTR and BMI1 subpopulation was detected in all NHOM samples, and 90% of OD samples, but only in 58.2% of the OSCC samples (Figure 4C, Supplementary Figure S3D). It is worth mentioning here that out of the OSCC samples that showed no co-localization between p75NTR and BMI1 (n = 74), 51 samples (68.9%) and 13 samples (17.6%) had very low percentage of positive cells for p75NTR and BMI1, respectively, meaning that this co-localization was detected mostly in samples with high scores of the two markers. On the other hand, co-localization of ALDH1A1 and BMI1 was found to be a more frequent finding in OD samples, but the finding was not statistically significant (Figure 4D, Supplementary Figure S3E). The frequency of this co-localization was found not to be associated with any of the clinical parameters investigated.

Cells positive for both stem cell markers p75NTR and ALDH1A1 and the self-renewal marker BMI1 were detected within basal layer cells of 64.5% of NHOM, 50% of ODs and only 19.2% of OSCC (Figure 4E, Supplementary Figure S3F). Out of the OSCC samples that showed co-localization (19.2%, n = 34), three cases (8.8%) showed co-localization of the three markers in more than 50% of the tumor cells (Supplementary Figure S3F). No statistically significant association with any of the clinical parameters was found.

### 3.7. p75NTR+ Cells Displayed Higher Proliferative Activity than ALDH1A1 in NHOM and OSCC

**Figure 5:**
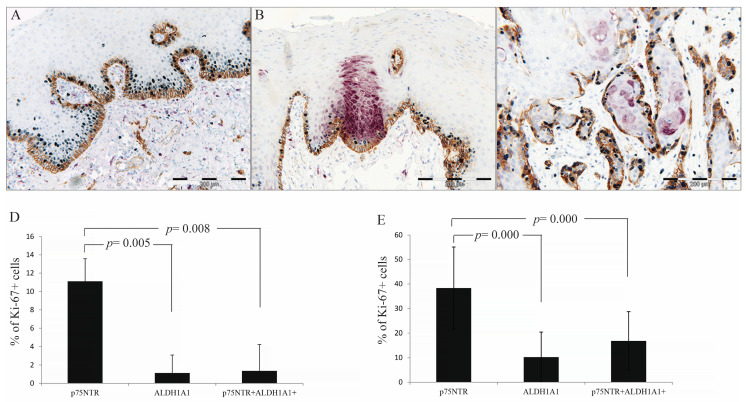
Quantification of proliferative CSC-marker-expressing cells. Formalin-fixed, paraffin-embedded tissues were subjected to triple IHC for p75NTR (brown), ALDH1A1 (purple) and Ki-67 (Dark grey). Panels A–C show representative images for NHOM (**A**), para-tumor epithelium (**B**) and OSCC (**C**). Panels D–E represent bar charts comparing the proportions of Ki-67+ cells within p75NTR+, ALDH1A1+, and double-positive p75NTR+ALDH1A1+ subpopulations in NHOM (**D**) and OSCC (**E**).

The mean percentages of Ki-67+ cells within p75NTR+, ALDH1A1+ and double-positive p75NTR+ALDH1A1+ subpopulations were compared in samples showing detectable expression of both CSC markers [NHOM (n = 19) and OSCC samples (n = 58)] (Figure 5A–C). Friedman test revealed significant differences in proliferative rates among the three cell subpopulations in both NHOM (*p* = 0.006) and OSCC (*p* < 0.001), with the p75NTR+ cells consistently showing the highest percentage of Ki-67 nuclei (Figure 5). Pairwise comparison using Wilcoxon-Rank test showed a significant difference between proliferative p75NTR+ and ALDH1A1+ cell subpopulations in NHOM (*p* = 0.005) and OSCC (*p* = 0.000) as well as between proliferative p75NTR+ and p75NTR+ALDH1A1+ subpopulations in NHOM (*p* = 0.008) and in OSCC (*p* < 0.001). No difference was observed between proliferative ALDH1A1+ and p75NTR+ALDH1A1+ cell subpopulations in either NHOM (*p* = 0.217) or OSCC (*p* = 0.273) (Figure 5D,E).

### 3.8. Comparable Expression of p75NTR, ALDH1A1, and BMI1 Between Tumor Center, Invading Front, and Lymph Node Metastasis

Immunohistochemical scores for the CSC markers and BMI1 in the tumor center were compared to those obtained at the tumor invading front (n = 25) and in paired metastatic lymph node tumor islands (n = 19). There were no statistically significant difference observed for any of the three markers investigated (Supplementary Figure S5A–D and Table S4).

In addition, mRNA expression levels of p75NTR and ALDH1A1 were analyzed in laser-microdissected samples from the tumor center and tumor invading front in OSCC samples (n = 23). Due to limited RNA yield, valid pairwise comparisons were possible in only seven samples for ALDH1A1 and eight for p75NTR. Consistent with the IHC data, no statistically significant differences were detected at the mRNA level (Supplementary Figure S5E).

### 3.9. Increased Heterogeneity of CSC Marker Expression with Cancer Progression In Vitro

**Figure 6:**
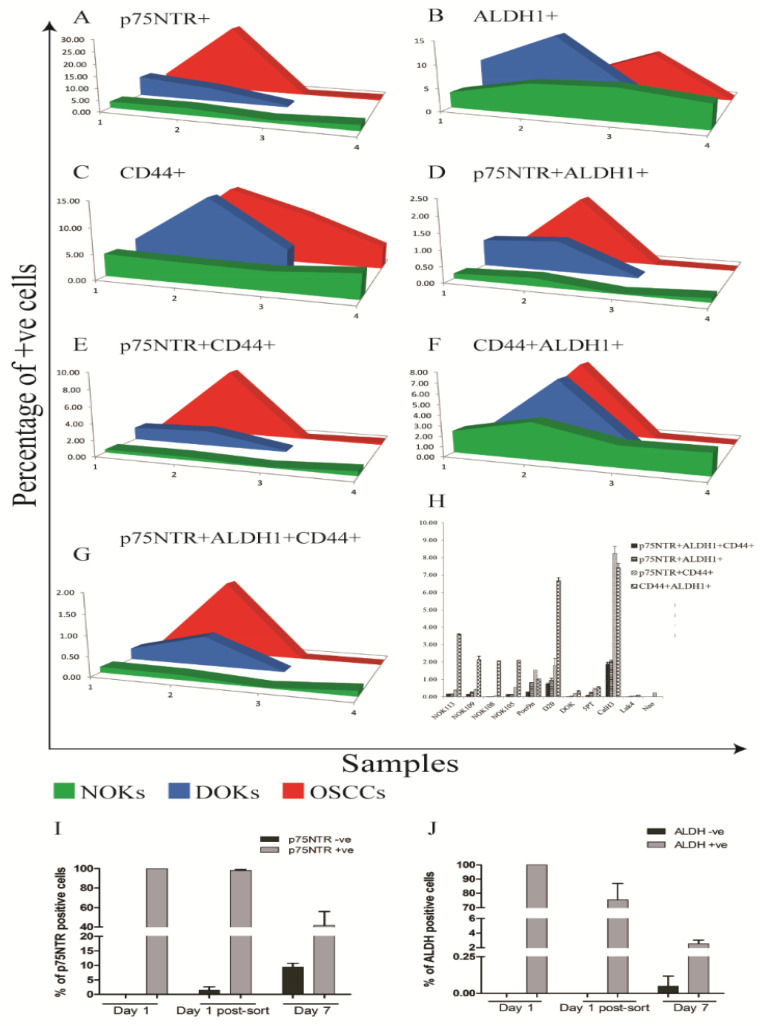
Co-staining and marker overlap. Area charts representing the results of multiple FACS analysis in NHOM, OD and OSCC-derived cells (**A**–**H**). Each marker showed low frequencies in NHOM (**A**), slightly higher in OD (**B**), and markedly variable among OSCC lines (**C**). Double co-positive fractions p75NTR^+^ALDH1^+^ (**D**), p75NTR^+^CD44^+^ (**E**), CD44^+^ALDH1^+^ (**F**), and triple co-positive fractions p75NTR^+^ALDH1^+^CD44^+^ (**G**) were consistently rare. De novo generation of p75NTR+ and ALDH1A1+ CalH3 cells (**I**,**J**). Error bars represent SD.

When expression of p75NTR, ALDH1 and CD44 was analyzed simultaneously by FACS (Figure 6, Supplementary Figure S1) of cell lines and primary cells comprising an in vitro progression model of OSCC [43], a pattern similar to that identified by IHC was detected. FACS analysis showed that expression levels of p75NTR, ALDH1 and CD44 displayed a wider range in OD and OSCC cells as compared to NOKs. Co-positive double fractions (p75NTR+ALDH1+, p75NTR+CD44+, CD44+ALDH1+) and co-positive triple fractions of p75NTR+ALDH1+CD44+ were consistently rare, generally <1% in NHOM and OD, and <2% in OSCC cells. The highest overlaps occurred in the CaLH3 line (2.0% p75NTR+ALDH1+; 8.3% p75NTR+CD44+; 7.4% CD44+ALDH1+; 1.9% triple-positive). The overall pattern shows minimal overlap between markers, with each defining largely independent cell fractions (Figure 6, Supplementary Tables S5 and S6).

ALDH1^High^ and p75NTR^High^ CaLH3 cells sorted by FACS displayed distinct gene expression profiles for CSC-related genes when analyzed by qRT-PCR. Significantly higher mRNA levels of previously suggested CSC markers (CD44, PDPN encoding Podoplanin, POU5 F1 encoding the transcription factor Oct4A) were detected on p75NTR^High^ cells in comparison to p75NTR^Low^ (Supplementary Figure S6). This pattern was the opposite in cells sorted according to ALDH1 expression. Instead, vimentin expression was seen, which indicates epithelial-to-mesenchymal transition was higher in ALDH1^High^ cells when compared to ALDH1^Low^ cells. Notably, the qRT-PCR results showed higher expression of epithelial differentiation markers (IVL and KRT10) by ALDH1^High^ as compared to ALDH1^Low^ cells, while the opposite was observed for cells sorted for p75NTR expression. This indicates a less differentiated phenotype for the p75NTR^High^ cells compared to ALDH1^High^ cells.

### 3.10. Distinct Kinetics of ALDH1 and p75NTR Expression in OSCC-Derived Cells Propagated In Vitro

OSCC-derived cells sorted for p75NTR or ALDH1 expression were propagated in culture for a week, and FACS analysis was performed as before. Based on the concomitant isotype control, cells propagated from the sorted p75NTR^Low^ cells contained 8.06% p75NTR^High^ cells after one-week propagation in culture, while p75NTR^High^-sorted subpopulation was found to contain 59.9% p75NTR^High^ cells after the same period in culture (Figure 6I). Assessment of ALDH1 activity after one-week propagation in culture showed that cells propagated from ALDH1^Low^ cells contained 0.17% ALDH1^High^ cells, while cells propagated from ALDH1^High^ subpopulation contained only 3.99% ALDH1^High^ cells (Figure 6J, Supplementary Figure S7). These data show that expression of these two stem cell markers has a complex and differential regulation and indicates that p75NTR^High^ and ALDH1^High^ OSCC-derived cells can arise de novo with different kinetics.

## 4. Discussion

One of the key findings of this work was the marked heterogeneity in the expression of the two CSC markers ALDH1 and p75NTR in OD and OSCC as compared to NHOM. This corroborates previous findings on CSC markers from both OSCC [15,40,44], as well as other cancer models. In OSCC, CD44+ cells varied from 0.1% to 41.72% in a previous study [15]. In acute myeloid leukemia, the frequency of ALDH1+ cells was found to vary from 1 to 16% [45], and another study found that CD34+CD38− varied by 1000-fold in a cohort of 16 patients [46]. Of note, we also found that the co-expression of the two markers by OSCC cells was an infrequent finding in both OSCC patient material as well as in OSCC-derived cell lines. Although some OSCC samples were completely lacking the expression of the two markers, significantly higher frequency of positive cells was detected in OSCC and OD when compared to NHOM. This study shows, for the first time to our knowledge, that multiple cell subpopulations expressing different stem cell markers co-exist in both normal and neoplastic oral mucosa and that the consequent inter-patient CSC heterogeneity increases with malignant progression. Interestingly, we could detect some OD and OSCC cases in which not all the CSC phenotypes were present. We could identify several co-existent stem cell phenotypes in all NHOM samples and NOK cell strains, although with much lower frequency and co-localization, while in most of the OSCC samples and cell lines, only one stem cell phenotype seemed to be dominant at a certain time point. This phenomenon could be observed already in OD samples. The fact that some OSCCs were negative for both of the CSC markers investigated in this study indicates also that a CSC subpopulation with a different phenotype could have occurred with cancer progression in those particular cases, different from the stem cell phenotype of the original normal mucosa. In addition, the absence of the self-renewing marker BMI1 and of both CSC markers in a subset of OSCC (n = 5) suggests that progression of these particular tumors may be driven by an alternative cellular phenotype independent of BMI1 involvement. This finding implies that BMI1 might not be indispensable for maintaining a stem cell hierarchy, or that such tumors might not adhere to a hierarchical stem cell model [7].

p75NTR+ cells exhibited a higher proliferation rate than ALDH1+ cells. As a member of the TNF receptor superfamily, p75NTR is capable of mediating a wide range of cellular events from apoptosis to proliferation, depending on factors such as its level of expression, post-translational modifications, dimerization state, ligand binding, and interactions with specific co-receptors and intracellular partners [47,48,49,50]. However, a role of this receptor in promoting survival of oral and esophageal keratinocyte stem cells has been suggested [16,51]. Additionally, a role in promoting proliferation in mouse embryonic stem cells was also reported [52]. Self-renewal, evidenced by expression of BMI1, was also a consistent finding in p75NTR+ cells in all samples. This is in agreement with our previous findings that an oncogene FOXM1, which could transactivate BMI1, promoted the clonogenicity of primary oral human keratinocytes expressing high levels of p75NTR^High^ but not in p75NTR^Low^ cells [53,54]. The association between p75NTR and less differentiated OSCC cells, generally believed to be associated with poor prognosis [55] and to include CSCs [44], points to the p75NTR+ subpopulation as a population of cells enriched for cells with stem cell properties. In vitro and in vivo analysis of p75NTR^High^ and p75NTR^Low^ OSCC cells supports the finding that this marker is associated with a group of less differentiated OSCC cells [22]. A possible biological mechanism by which P75NTR drives OSCC progression stems from its role in modulating voltage-gated sodium channels in excitable cells such as nerves and muscles [56,57]. These channels have been shown to be present in poorly differentiated OSCC and were shown to drive cancer cell proliferation and metastasis [58,59]. Future research could focus on this avenue as a potential therapeutic target in OSCC. Notably, a markedly high frequency of positive cells (more than 50% p75NTR+ cells) was found in 15.8% of OSCCs and in one OSCC-derived cell line (CaLH3) but not in any NHOM or in any of the NOK strains analyzed. Our results also showed an association between the frequency of p75NTR+ cells and poorer survival of patients with T1 and T2 tumors. This suggests a significance of the presence of p75NTR+ cells in that group of patients, and it is in line with previous publications that have also reported the significance of p75NTR in prostate [60] and pancreatic [61] cancers, melanoma [18], oral [21], oesophageal [19,62,63], and laryngeal squamous cell carcinoma [64]. This might indicate that the p75NTR+ population is only important at early tumor stages, while larger tumors can progress without input from this cell subpopulation.

High expression of ALDH1A1 was detected in OD patient material and derived cell lines, as well as in the pre-tumor epithelium of OSCC patients. High expression of ALDH1A1 was found to be associated with higher likelihood of lymph node metastasis. The clone-like distribution pattern of ALDH1A1-expressing cells was not found to be associated with clinical information or patient survival, but significantly high frequency of ALDH1A1+ cells was detected in an OD-derived cell line by flow cytometry (Figure 6B). Together with the association of lymph node metastasis, and the similarity between the tumor center and lymph node metastasis, it is plausible that ALDH1A1-expressing cells might play a role in invasion as suggested before in inflammatory breast cancer [65] or metastasis as suggested before in OSCC [66]. Of note, ALDH1+ cells were suggested before as being the CSC undergoing EMT, and in this line we also found ALDH1^High^ cells to express higher levels of vimentin than the p75NTR^High^ cells. However, our qRT-PCR results also showed a higher expression of epithelial differentiation markers by ALDH1^High^ as compared to p75NTR^High^, indicating a less differentiated phenotype for the p75NTR^High^ cells compared to ALDH1^High^ cells. p75NTR+ cells were also suggested previously to be the feeding reservoir of the tumor cells and the self-renewal compartment [53].

Notably, all subpopulations of cells sorted either for ALDH1 or for p75NTR, high or low expression, showed that they can regenerate from each other. The fact that the subpopulation enriched for stem cells such as the ALDH1^High^ or p75NTR^High^ cells generate non-stem cells was expected and fits well in the stem cell hierarchy theory, but the ability of the subpopulations depleted for CSC markers (ALDH1^Low^ or p75NTR^Low^) to generate de novo subpopulations highly expressing these CSC markers (ALDH1^High^ or p75NTR^High^ cells) was unexpected. The dynamism we observed here is in line with the recent suggestions from research on CD44^High^ and CD44^Low^ cells isolated from breast cancers that displayed a contextually highly regulated equilibrium between stem cell-like cells and transit-amplifying neoplastic progenitors [67]. One can theorize that this equilibrium might have been disturbed in our case by the removal of one CSC phenotype and then the natural tendency to regain the equilibrium between CSC phenotypes, or with the non-CSC compartment inducing the highly plastic cancer cells to regenerate the missing CSC phenotype.

The low level of co-localization, both in patient samples and in cell lines, indicates also that p75NTR+ and ALDH1A1+ cells comprise two separate subpopulations with minimal overlap in OSCC. Although partial overlap between the two subpopulations was detected in some OSCC samples, no effect of the presence of this subpopulation was detected by looking into the clinical variables of the patients. The observed statistical association between ALDH1A1+ cells and lymph node metastasis, and between p75NTR+ cells and shorter survival of patients with favorable prognosis, suggests that CSC subpopulation in OSCC is a highly dynamic subpopulation showing different phenotypes at different time points of tumor progression, probably with different clinical connotations.

## 5. Conclusions

Taken together, the data presented here demonstrate the intra- and inter-tumor phenotypic diversity of CSC. This raises novel challenges in terms of the use of CSC markers as predictive biomarkers and indicates that a more personalized approach is needed for their use as an efficient therapeutic target for oral squamous cell carcinoma, since every OSCC might have its specific CSC phenotype, and some might not follow a stem cell hierarchy at all.

## Data Availability

Data can be obtained upon reasonable request to the corresponding author.

## Supplementary Materials

The following supporting information can be downloaded at: https://www.mdpi.com/article/10.3390/cancers17213547/s1, Figure S1: Density plots illustrating analysis of FACS data of LuC4 cells for all three markers; Figure S2: Histogram showing survival time of the study participants (A). Kaplan–Meier’s curves showing difference in survival probabilities between patients according to different demographic and clinical information (B–F); Figure S3: Heat Maps illustrating the IHC scores for each of the samples individually. Samples are in the same order in all heat maps (A–F); Figure S4: Comparison of the clinical variables between patients scoring High or Low in IHC for p75NTR and ALDH1A1; Figure S5: FFPE OSCC tissues subjected for triple IHC for p75NTR (purple), ALDH1A1 (gray) and BMI1 (brown). Tumor center (A) and invading front (B) from same patient. Tumor center (C) and lymph node metastasis (D) from same patient. Comparison of the relative mRNA expression of the two CSC markers between tumor center and invading front from a laser-microdissected FFPE samples (E), error bars represent SD; Figure S6: qRT-PCR of FACS sorted CalH3 cells for CSC (A) and differentiation markers (B); Figure S7: density plots illustrating FACS of CalH3 cells for ALDH1 at Day 1 (A–C), and ALDH1^High^ (D,E) and ALDH1^Low^ (F,G) cells after propagation in culture. Table S1: Scores used to quantify IHC of the studied CSC markers and their co-localization; Table S2: Patients demographic and clinical information cross-tabulated against survival; Table S3: Cox regression hazard models to investigate the effect of tumor size on p75NTR as an independent predictor of survival; Table S4: Comparison of the IHC scores between tumor center and invading front or lymph nodes metastasis; Table S5: Results of the multiple FACS analysis of NHOM, OD and OSCC-derived cells; Table S6: Results of the multiple FACS analysis (continued).
