# Supplementary material for: Identification of Phenotypically Distinct Cancer Stem Cell Subpopulations in Oral Squamous Cell Carcinoma
Source: Cancers (Basel). 2025 Nov 1;17(21):3547. doi: 10.3390/cancers17213547 (PMC12606748; doi:10.3390/cancers17213547)

Supplementary Material

Supplementary Table S1: Scores used to quantify IHC of the studied CSC markers and their co-localization.

score	p75NTR	ALDH1A1	BMI1	Co-localization
0	<1%	<5%	<1%	0%
1	1-25%	5-25%	1-25%	>0, <25%
2	26-50%	25-50%	26-50%	26-50%
3	>50%	>50%	>50%	>50%

Supplementary Table S2: Patients demographic and clinical information cross-tabulated against survival.

	Patient overall survival (N = 177)				
	N= < 5 years	N= 5 to 10 years	N= > 10 years	N= Total	X^2 P-value
**Total (%)**	96 (54.2%)	48 (27.1%)	33 (18.6%)	177 (100.0%)	
**Gender N**					
Female	37 (38.5%)	17 (35.4%)	18 (54.5%)	72 (40.7%)	0.186
Male	59 (61.5%)	31 (64.6%)	15 (45.5%)	105 (59.3%)	
Total	96 (100.0%)	48 (100.0%)	33 (100.0%)	177 (100.0%)	
**Age**					
21-30	1 (1.0%)	0 (0.0%)	1 (3.0%)	2 (1.1%)	0.009 *
31-40	0 (0.0%)	0 (0.0%)	2 (6.1%)	2 (1.1%)	
41-50	10 (10.4%)	3 (6.3%)	6 (18.2%)	19 (10.7%)	
51-60	20 (20.8%)	10 (20.8%)	14 (42.4%)	44 (24.9%)	
61-70	26 (27.1%)	17 (35.4%)	8 (24.2%)	51 (28.8%)	
71-80	21 (21.9%)	11 (22.9%)	1 (3.0%)	33 (18.6%)	
81-90	16 (16.7%)	7 (14.6%)	1 (3.0%)	24 (13.6%)	
91-100	2 (2.1%)	0 (0.0%)	0 (0.0%)	2 (1.1%)	
Total	96 (100.0%)	48 (100.0%)	33 (100.0%)	177 (100.0%)	
**Site**					
Tongue	46 (47.9%)	25 (52.1%)	21 (63.6%)	92 (52.0%)	0.262
Buccal mucosa	6 (6.3%)	5 (10.4%)	2 (6.1%)	13 (7.3%)	
Lip	0 (0.0%)	4(8.3%)	1(3.0%)	5 (2.8%)	
Gingiva	12 (12.5%)	3 (6.3%)	1 (3.0%)	16 (9.0%)	
Floor of mouth	19 (19.8%)	7 (14.6%)	5 (15.2%)	31 (17.5%)	
Overlapping	5 (5.2%)	2 (4.2%)	1 (3.0%)	8 (4.5%)	
Alveolar rim	8 (8.3%)	2 (4.2%)	2 (6.1%)	12 (6.8%)	
Total	96 (100.0%)	48 (100.0%)	33 (100.0%)	177 (100.0%)	
**TNM stage**					
Early: stage1	9 (10.1%)	12 (26.7%)	12 (36.4%)	33 (19.8%)	0.001 *
stage2	18 (20.2%)	13 (28.9%)	9 (27.3%)	40 (24.0%)	
Late: stage3	18 (20.2%)	11 (24.4%)	3 (9.1%)	32 (19.2%)	
stage4	44 (49.4%)	9 (20.0%)	9 (27.3%)	62 (37.1%)	
Total	89 (100.0%)	45 (100.0%)	33 (100.0%)	167 (100.0%)	
**Tumor size**					
Small size: T1	13 (14.4.5%)	13 (28.9%)	14 (42.4%)	40 (23.8%)	0.001 *

T2	33 (36.7%)	19 (42.2%)	12 (36.4%)	64 (38.1%)	
Large size: T3	12 (13.3%)	9 (20.0%)	3 (9.1%)	24 (14.3%)	
T4	32 (35.6%)	4 (8.9%)	4 (12.1%)	40 (23.8%)	
Total	90 (100.0%)	45 (100.0%)	33 (100.0%)	168(100.0%)	
**Lymph nodes**					
No metastasis	47 (53.4%)	27 (60.0%)	24 (72.7%)	98 (59.0%)	0.155
metastasis	41 (46.6.3%)	18 (40.0%)	9 (27.3%)	68 (41.0%)	
Total	88 (100.0%)	45 (100.0%)	33 (100.0%)	166 (100.0%)	
**Tumor differentiation**					
Low: Poor	10 (10.4%)	4 (8.3%)	1 (3.1%)	15 (8.5%)	0.334
Moderate	50 (52.1%)	21 (43.8%)	13 (40.6%)	84 (47.7%)	
High: High	36 (37.5%)	23 (47.9%)	18 (56.3%)	77 (43.8%)	
Total	96 (100.0%)	48 (100.0%)	32 (100.0%)	176 (100.0%)	
**Tumor recurrence**					
no recurrence	71 (74.0%)	35 (72.9%)	27 (81.8%)	133 (75.1%)	0.611
recurrence	25 (26.0%)	13 (27.1%)	6 (18.2%)	44 (24.9%)	
Total	96 (100.0%)	48 (100.0%)	33 (100.0%)	177 (100.0%)	

Supplementary Table S3: Cox regression hazard models to investigate the effect of tumor size on p75NTR as an independent predictor of survival.

Model	Overall sig.	covariate	Co-efficient	Sig.	Exp(B)	HR
**Analysis 1** (N= 177)						
**Crude**	0.247	p75NTR	-0.213	0.248	0.808	1.24
**Splitting data by Tumor size**						
T1 & T2	0.088	p75NTR	-0.418	0.09	0.658	1.52
T3 & T4	0.966	p75NTR	-0.015	0.966	1.015	1.03
**Adjusted for tumor size**	0	p75NTR	-0.4	0.104	0.67	1.49
		Tumor size	0.087	0.091	1.796	1.796
		Interaction	0.862	0.403	1.407	1.407
**Analysis 2** (N=144)						
**Crude**	0.015	p75NTR	-0.468	0.016	0.626	1.58
**Splitting data by Tumor size**						
T1 & T2	0.002	p75NTR	-0.918	0.002	0.441	2.23
T3 & T4	0.927	p75NTR	-0.032	0.927	0.969	1.03
**Adjusted for tumor size**	0	p75NTR	-0.82	0.002	0.44	2.27
		Tumor size	0.087	0.807	1.091	1.1
		Interaction	0.862	0.045	2.368	2.4

Supplementary Table S4: Comparison of the IHC scores between tumor center and invading front or lymph nodes metastasis.

			Tumor center			
			Low	High	Total	*p*-value
	ALDH1A1					
Invading front	ALDH1A1	Low	19 (100%)	5 (100%)	24 (100%)	ND
		High	0	0	0	
	p75NTR					
	p75NTR	Low	15 (88.2%)	6 (85.7%)	21 (87.5%)	0.289
		High	2 (11.8%)	1 (14.3%)	3 (12.5%)	
	BMI1					
	BMI1	Low	5 (100%)	5 (26.3%)	10 (41.7%)	0.074
		High	0 (0.0%)	14 (73.7%)	14 (58.3%)	
	ALDH1A1					
Lymph node metastasis	ALDH1A1	Low	13 (100%)	3 (50.0%)	16 (84.2%)	0.248
		High	0 (0%)	3 (50.0%)	3 (15.8%)	
	p75NTR					
	p75NTR	Low	13 (86.7%)	1 (25.0%)	14 (73.7%)	1.000
		High	2 (13.3%)	3 (75.0%)	5(26.3%)	
	BMI1					
	BMI1	Low	3 (75.0%)	5 (33.3%)	8 (42.1%)	0.221
		High	1 (25.0.3%)	10 (66.7%)	9 (57.9%)	

Supplementary Table S5: Results of the multiple FACS analysis of NHOM, OD and OSCC-derived cells.

	p75NTR+	ALDH1+	CD44+
NOK113	2.70 ± 0.00	6.57 ± 0.12	4.40 ± 0.78
NOK109	2.93 ± 0.23	7.03 ± 0.40	3.97 ± 0.25
NOK108	1.2 ± ND	3.4 ± ND	3.7 ± ND
NOK105	2.5 ± ND	5.2 ± ND	4.7 ± ND
Poe9n	8.5 ± ND	8.4 ± ND	5.1 ± ND
D20	5.7 ± 0.00	14.8 ± 0.14	14.45 ± 0.49
DOK	0.75 ± 0.07	3.35 ± 0.07	5.1 ± 0.14
5PT	5.3 ± 0.53	3.23 ± 0.06	3.17 ± 0.21
CaLH3	29.13 ± 0.55	9.7 ± 0.17	14.67 ± 0.49
LuC4	0.25 ± 0.07	3.85 ± 0.07	10.15 ± 0.21
Neo	0.7 ± ND	0 ± ND	4.4 ± ND

Supplementary Table S6: Results of the multiple FACS analysis (continued).

	p75NTR+ALDH1+	p75NTR+CD44+	CD44+ALDH1+	p75NTR+ALDH1+CD44+
NOK113	0.16 ± 0.01	0.37 ± 0.02	3.59 ± 0.04	0.14 ± 0.01
NOK109	0.25 ± 0.04	0.41 ± 0.03	2.13 ± 0.21	0.12 ± 0.02
NOK108	0.02 ± ND	0.07 ± ND	2.07 ± ND	0.01 ± ND
NOK105	0.14 ± ND	0.56 ± ND	2.10 ± ND	0.14 ± ND
Poe9n	0.83 ± ND	1.54 ± ND	1.04 ± ND	0.28 ± ND
D20	0.97 ± 0.10	1.82 ± 0.38	6.68 ± 0.16	0.74 ± 0.05
DOK	0.03 ± 0.00	0.17 ± 0.02	0.29 ± 0.06	0.01 ± 0.00
5PT	0.23 ± 0.03	0.45 ± 0.04	0.53 ± 0.06	0.07 ± 0.01
CaLH3	2.03 ± 0.08	8.25 ± 0.40	7.42 ± 0.24	1.88 ± 0.10
LuC4	0.01 ± 0.01	0.03 ± 0.01	0.09 ± 0.01	0.00 ± 0.00
Neo	0 ± ND	0.23 ± ND	0 ± ND	0 ± ND

Supplementary Figure S1: Density plots illustrating analysis of FACS data of LuC4 cells for all three markers.

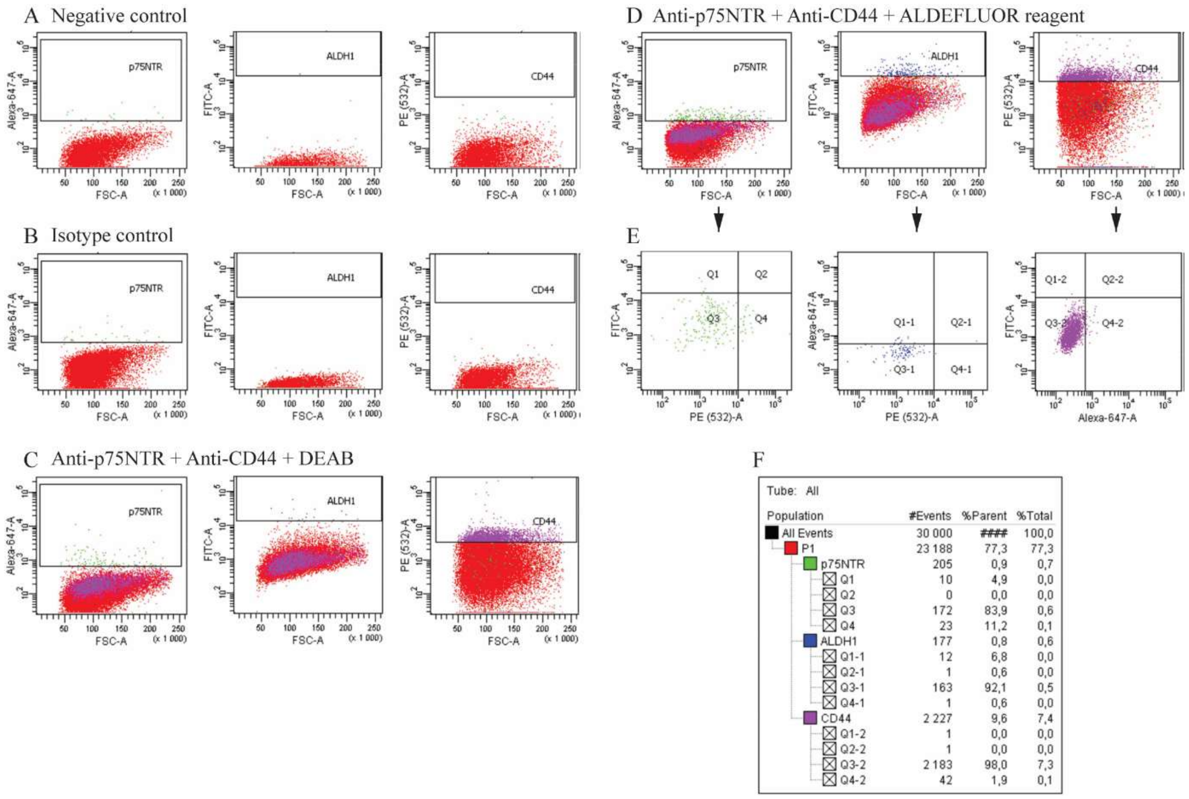

Supplementary Figure S2: Histogram showing survival time of the study participants (A). Kaplan-Meier`s curves showing difference in survival probabilities between patients according to different demographic and clinical information (B-F).

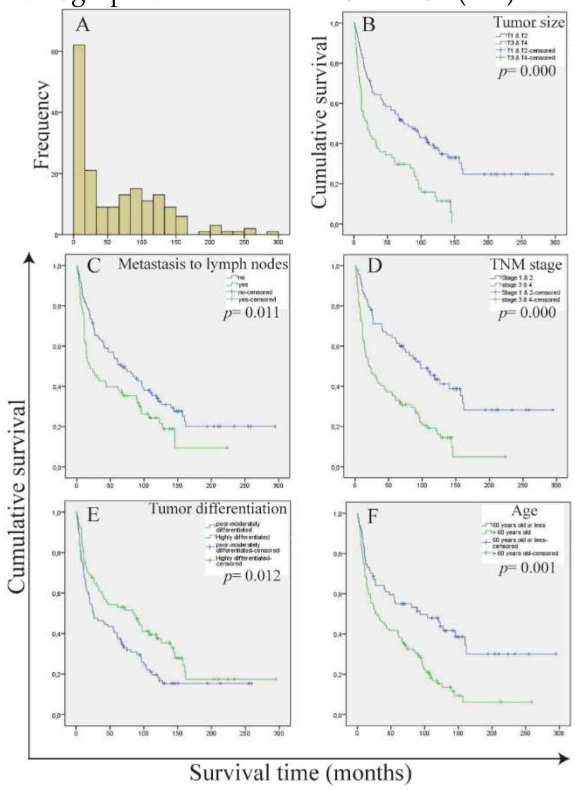

Supplementary Figure S3: Heat Maps illustrating the IHC scores for each of the samples individually. Samples are in the same order in all heat maps (A-F).

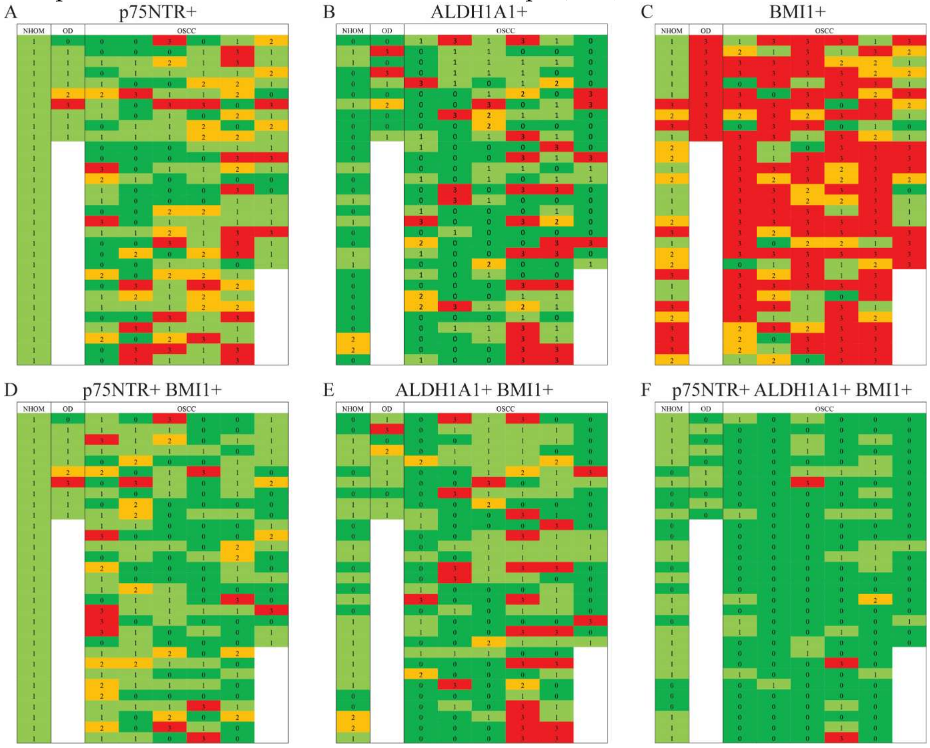

Supplementary Figure S4: Comparison of the clinical variables between patients scoring High or Low in IHC for p75NTR and ALDH1A1.

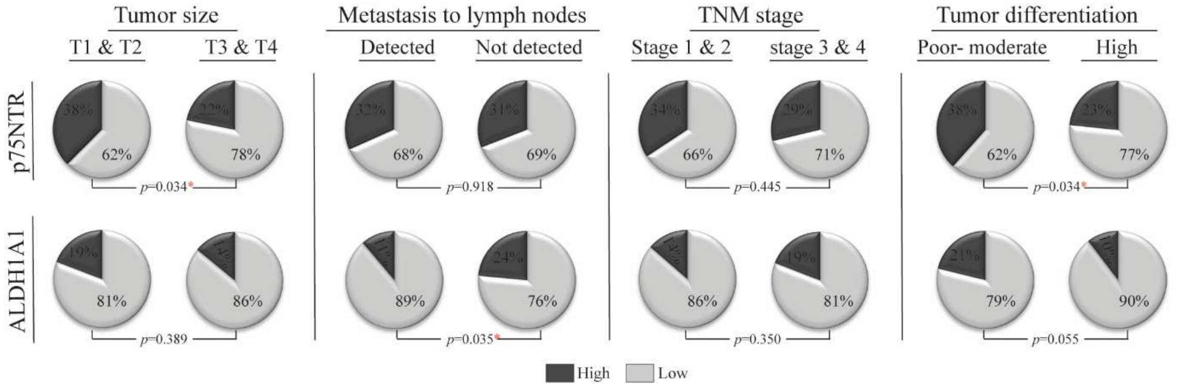

Supplementary Figure S5: FFPE OSCC tissues subjected for triple IHC for p75NTR (purple), ALDH1A1 (gray) and BMI1 (brown). Tumor center (A) and invading front (B) from same patient. Tumor center (C) and lymph node metastasis (D) from same patient. Comparison of the relative mRNA expression of the two CSC markers between tumor center and invading front from a laser micro-dissected FFPE samples (E), error bars represent SD.

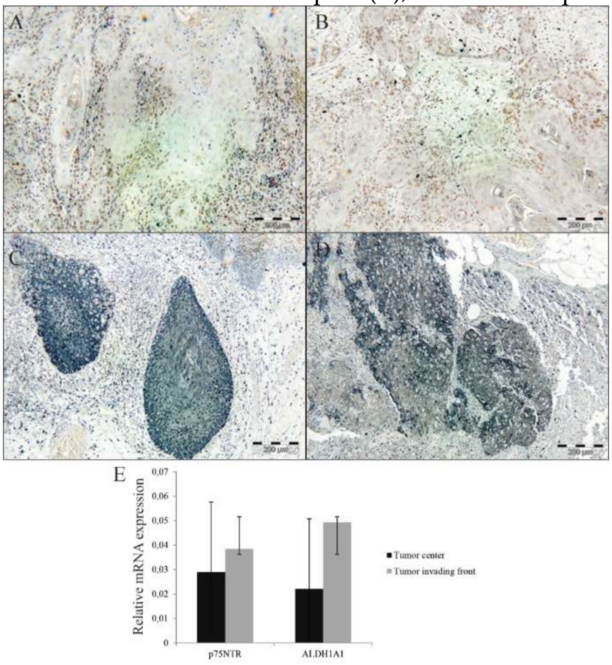

Supplementary Figure S6: qRT-PCR of FACS sorted CalH3 cells for CSC (A) and differentiation markers (B)

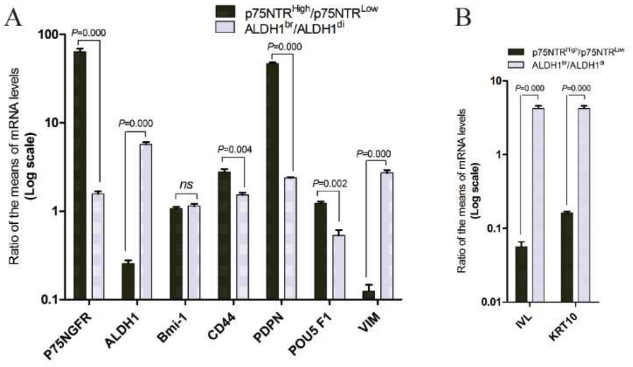

Supplementary Figure S7: density plots illustrating FACS sorting of CalH3 cells for ALDH1 at Day 1 (A-C), and ALDH1^{High} (D,E) and ALDH1^{Low} (F,G) cells after propagation in culture.

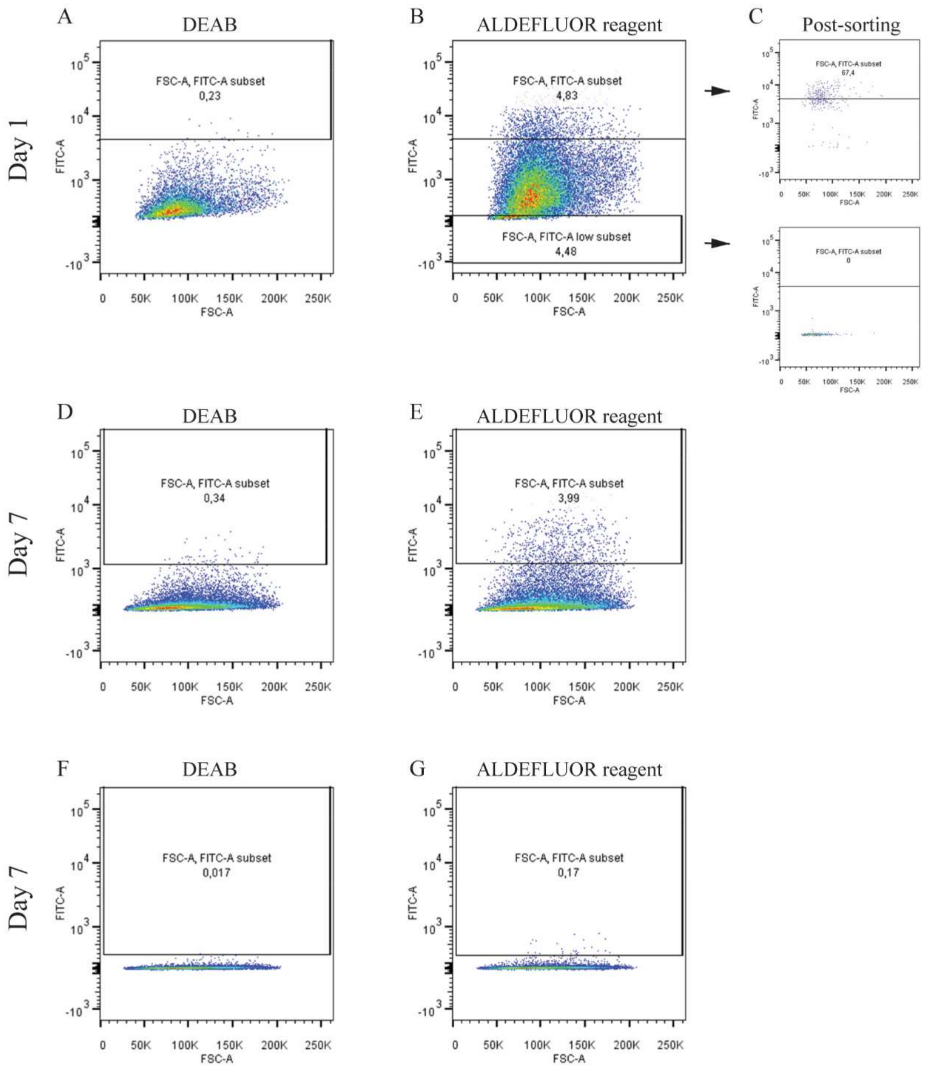

Supplement: Supplementary file 1 [file cancers-17-03547-s001.zip › cancers-3937464-supplementary.pdf]
